# Dipolar Order Induced Electron Spin Hyperpolarization

**DOI:** 10.1021/acs.jpclett.4c00294

**Published:** 2024-05-13

**Authors:** Asif Equbal, Chandrasekhar Ramanathan, Songi Han

**Affiliations:** † Department of Chemistry, New York University Abu Dhabi, P.O. Box 129188, Abu Dhabi, United Arab Emirates; ‡ Center for Quantum and Topological Systems, New York University Abu Dhabi, P.O. Box 129188, Abu Dhabi, United Arab Emirates; § Department of Physics and Astronomy, Dartmouth College, Hanover, New Hampshire 03755, United States; ∥ Department of Chemistry and Biochemistry, University of California, Santa Barbara, Santa Barbara, California 93106, United States; ⊥ Department of Chemical Engineering, University of California, Santa Barbara, Santa Barbara, California 93106, United States

## Abstract

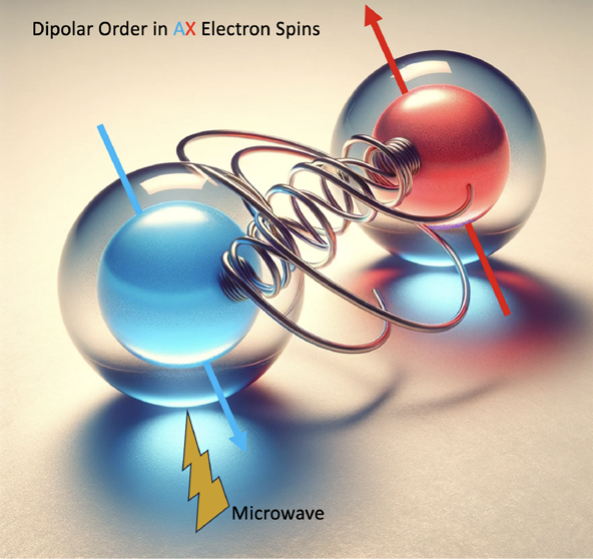

The structure of coupled electron spin systems is of fundamental interest to many applications, including dynamic nuclear polarization (DNP), enhanced nuclear magnetic resonance (NMR), the generation of electron spin qubits for quantum information science (QIS), and quantitative studies of paramagnetic systems by electron paramagnetic resonance (EPR). However, the characterization of electron spin coupling networks is nontrivial, especially at high magnetic fields. This study focuses on a system containing high concentrations of trityl radicals that give rise to a DNP enhancement profile of ^1^H NMR characteristic of the presence of electron spin clusters. When this system is subject to selective microwave saturation through pump–probe ELectron DOuble Resonance (ELDOR) experiments, electron spin hyperpolarization is observed. We show that the generation of an out-of-equilibrium longitudinal dipolar order is responsible for the transient hyperpolarization of electron spins. Notably, the coupled electron spin system needs to form an AX-like system (where the difference in the Zeeman interactions of two spins is larger than their coupling interaction) such that selective microwave irradiation can generate signatures of electron spin hyperpolarization. We show that the extent of dipolar order, as manifested in the extent of electron spin hyperpolarization generated, can be altered by tuning the pump or probe pulse length, or the interpulse delay in ELDOR experiments that change the efficiency to generate or readout longitudinal dipolar order. Pump–probe ELDOR with selective saturation is an effective means for characterizing coupled electron spins forming AX-type spin systems that are foundational for DNP and quantum sensing.

Electron spins play a crucial role in a wide scope of physical sciences. Specifically, the structure of coupled electron spin systems is of fundamental interest to many spectroscopic applications, electron paramagnetic resonance (EPR) spectroscopy for characterizing molecular assemblies, paramagnetic nuclear magnetic resonance (NMR), and dynamic nuclear polarization (DNP) amplified nuclear magnetic resonance (NMR). Furthermore, coupled electron spin systems are of growing interest in quantum information science (QIS) as potential qubits and quantum sensors.^1−5^

There are emerging studies in DNP that show the potential utility of using many-electron spin clusters including recent thermal mixing DNP studies at high magnetic fields.^6−11^ Here, the control over generating an isolated electron spin system or controlling the number of coupled electron spins in a system is critical to fully understand the DNP mechanism. For example, to differentiate between Overhauser Effect (OE) DNP,^12^ Solid Effect (SE) DNP,^13^ Cross Effect (CE) DNP,^14^ and Thermal Mixing (TM) DNP,^11^ we need to understand whether, and how many, coupled spins are responsible for the observed DNP process. Understanding the operational DNP mechanism will change the design principle to developing the next generation of polarizing agents (PAs) as well as the required DNP instrumentation. PAs that readily generate polarization transfer with low microwave power requirements can ease stringent experimental and instrumentation requirements.

In QIS, there are several notable efforts where optical excitation generates longitudinal dipolar order of coupled electron spins through the generation of radical pairs.^15−19^ For such a system to provide viable spin qubits, they need to be coupled yet separately addressable. This condition can be fulfilled if the generated electron spin pair corresponds to an AX type spin system.

However, the characterization of electron spin coupling is often nontrivial if there is strong coupling or a complex network of coupled electron spins, and if the spin Hamiltonian consists of both heterogeneous and homogeneous interactions. The focus of our study is to illustrate how to distinguish isolated and coupled electron spin systems at high magnetic fields. The relevant coupling for QIS and DNP often comes from electron–electron (e–e) distances of approximately 1 nm or even the subnanometer scale and hence evades techniques such as Double Electron Electron Resonance (DEER). This is because DEER cannot directly measure the dipolar coupling distribution in this range due to spectral overlap between pump and probe spin populations. Out-of-phase Electron Spin–Echo Envelope Modulation (ESEEM) is sensitive to shorter range distances, but this technique has been traditionally applied to nearly pure spin-correlated radicals generated by optical excitation.^20,21^ However, coupled electron spins, at thermal equilibrium under typical DNP conditions, do not have a correlated spin order. In this study, we seek to identify the existence of coupled electron spins using microwave pulse irradiation which transforms the electron Zeeman order to longitudinal dipolar order, i.e., a correlated spin order.^22^ This transformation can be done by adiabatic rotating frame transformation (ADRF),^23^ but the ADRF scheme cannot be readily implemented for fast relaxing electron spin systems at high magnetic field (commercial solid-state DNP operates at 9 T and higher, and our home-built setup at 7 T) for which the microwave sources available today typically cannot deliver coherent electron spin manipulation. In this study, we explore an alternative approach of using two frequency pump–probe pulsed EPR known as Electron–Electron Double Resonance (ELDOR) to uncover the response of an e–e coupling network under microwave irradiation. ELDOR as a means to study DNP mechanisms was demonstrated first by Vega and co-workers for the study of electron spectral diffusion in a coupled e–e network.^24^ The basic concept is to pump (excite) a subset of coupled electron spins to mimic the (partial) saturation effect of microwave irradiation under DNP conditions and subsequently probe (detect) the resulting electron spin polarization of the probed electron spins. The technique is similar to various saturation transfer magnetic resonance experiments. The depth, width, and spectral features of the electron spin saturation profile provide information about the coupling (direct and mediated through a network) between electrons resonating at the pump and probe frequencies.^25^

In this study, we focus on systems containing high concentrations of trityl or BDPA radicals as test systems that have been reported to form electron spin clusters and give rise to DNP enhancement of ^1^H NMR and signatures indicating that multiple coupled electron spins are involved in the transfer process.^8,26−28^ The DNP enhancement versus microwave (μw) irradiation frequency measured at 15 K and 6.9 T for trityl-OX063 vitrified (35 mM) in water–DMSO solution is shown in Figure 1. The DNP profile is acquired at two different μw powers, 100 and 350 mW. The dispersive features obtained at the center (marked with asterisk) is readily generated using low μw powers and is *a priori* unexpected in the sense that it cannot originate from the (expected) SE effect. The possible origin is the triple-flip transition, which is achieved when the EPR line is heterogeneously broadened beyond the (intrinsic) g-anisotropy, which can originate from multiple coupled electron spins. The underlying transfer process has been attributed to the thermal mixing DNP mechanism. The EPR line broadened by dipolar (D) and/or exchange (J) coupling between trityl electron spins can facilitate the triple-flip DNP.^11^ In particular, a coexistence of strongly coupled and weakly coupled electron spins is conducive to generating and maintaining a large electron polarization difference or gradient, Δ*P*_e_, across the EPR spectrum upon μw irradiation. A large Δ*P*_e_ spanning a frequency (equal to or larger than the nuclear Larmor frequency) is critical to enhancing nuclear polarization via the triple-flip mechanism.^7,29,30^ The low μw requirement to achieve a large Δ*P*_e_ in multielectron spin systems consisting of narrow line radicals has attracted attention because the low-power μw sources are much more versatile and affordable compared to the commonly used gyrotron setups for SE DNP. However, to use this principle to design new PAs, we need tools to directly probe the properties and response of the multielectron spin system to microwave irradiation. We turn to pump–probe ELDOR for this purpose.

**Figure 1:**
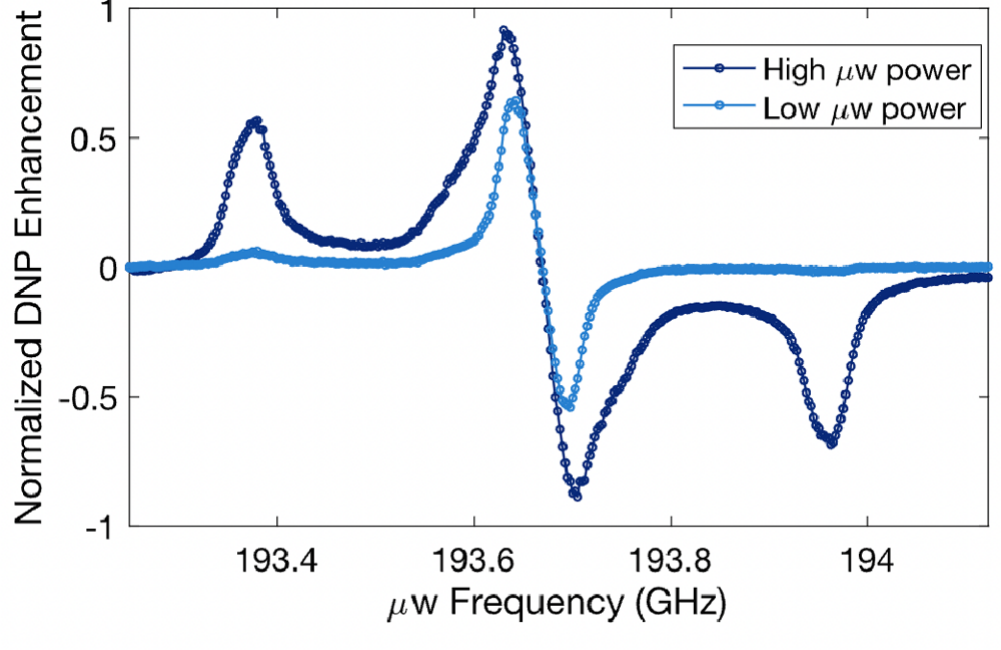
DNP enhancement as a function of μw frequency for two different powers: 350 mW (high) and 100 mW (low) at 6.9 T and 14 K under static conditions, measured for 35 mM trityl-OX063. At high power, a strong TM DNP is obtained at the central (dispersive) peak and an SE DNP at an offset of 294 MHz. In contrast, at low power, the TM DNP is still strong, while the SE DNP is almost negligibly small.

All EPR experiments are performed under the same conditions as the DNP experiments (shown in Figure 1). The first sample is the same 35 mM trityl-OX063 in DMSO–water, where the thermal mixing (TM) effect is very pronounced compared to solid effect DNP, and the electron spin relaxation times are long enough to readily perform EPR measurement with a home-built dual EPR-DNP setup at 6.9 T.^31^ Our goal is to determine the EPR signatures of a strongly coupling electron spin network in these samples.

The EPR spectrum was recorded using a solid echo pulse sequence and shows a broadened resonance line for the trityl radical spanning more than 150 MHz at the base, much beyond its g-anisotropy at 6.9 T that only gives rise to a 65 MHz line width (Figure 2A). The observed line broadening must be due to e–e coupling that in reality may even be broader than the apparent line width spanning 150 MHz at the base, given that fast relaxing electron spin populations that give rise to a broad baseline are not readily detected using the low-power microwave source available for 200 GHz.^8^ The echo-detected EPR line shape furthermore shows features characteristic of instantaneous diffusion according to a central “dip” in the EPR spectrum. Instantaneous diffusion, a phenomenon relevant for pulsed EPR experiments of relatively strongly dipolar coupled paramagnetic spin systems, is due to pulse-induced spin diffusion that occurs when pulses excite electron spins out of equilibrium of a similar resonance frequency that are dipolar coupled to each other, causing faster dephasing by altering each others’ resonance frequency by modulation of their local dipolar fields. This in turn attenuates echo intensity in a two-pulse detection scheme.^32^ Spectral packets with higher spin density (i.e., at the center) are more severely affected than those with lower spin density (i.e., the shoulders), resulting in a spectrum with a center whose amplitude decays faster in comparison with off-center spins. The prominent instantaneous diffusion implies that the spin systems constituting the EPR spectrum are strongly dipolar coupled.

**Figure 2:**
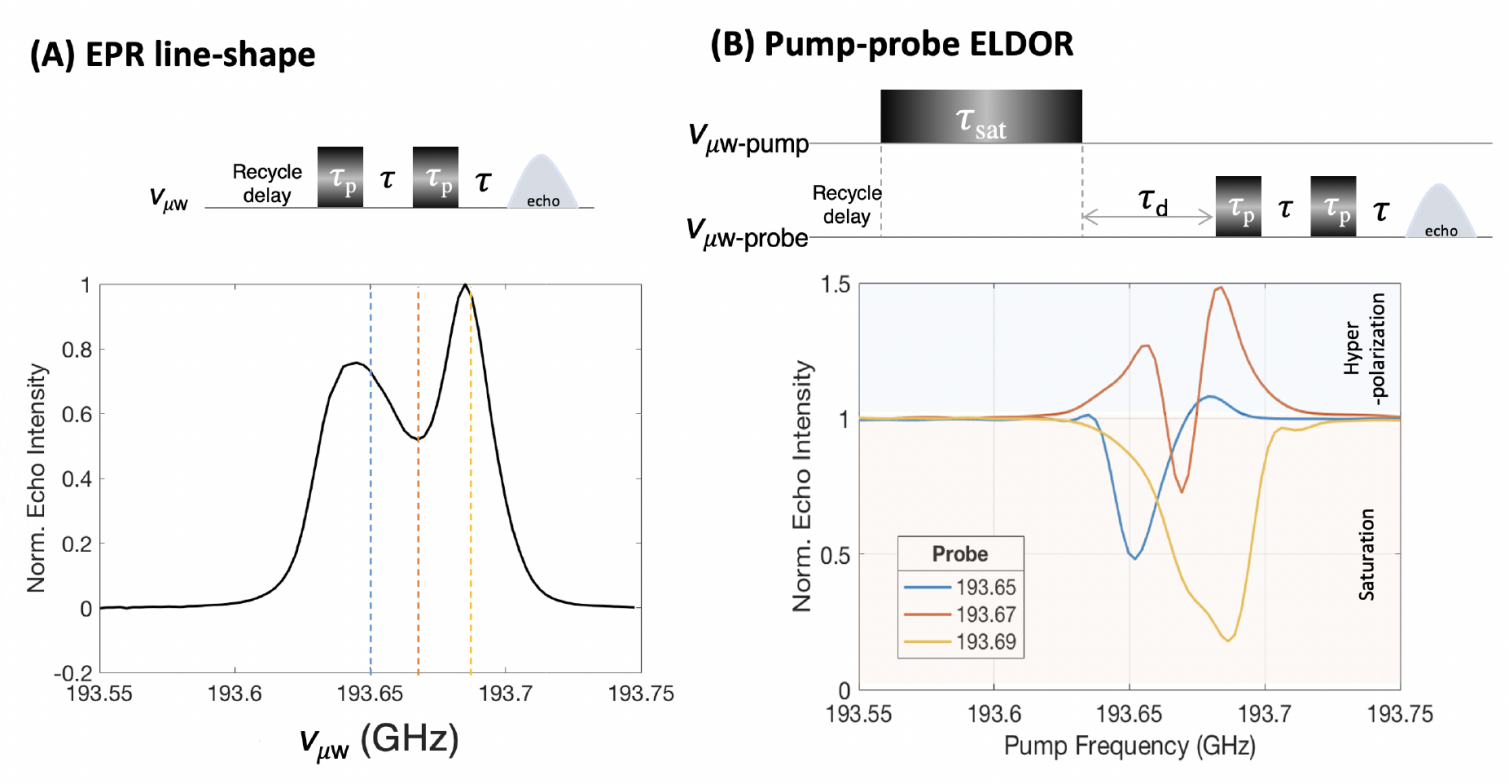
(A) Experimental EPR line shape of 35 mM trityl in DMSO–water solvent recorded using the shown pulse scheme at 15 K. (B) ELDOR polarization profile for three different probe frequencies marked with vertical lines in A.

As already mentioned, pulsed DEER cannot be used for the characterization of 35 mM trityl or other radicals at high concentrations in the several tens of mM range due to interference from cross talk, rapid relaxation and instantaneous diffusion.^1^ Instead, we relied on pump–probe ELDOR to characterize the coupling network of the electron spin system. We performed ELDOR while varying the pump frequency, pump power, probe frequency and probe length, as well as delay between pump and probe pulses. Despite the limited microwave irradiation capability at high (7 T) magnetic fields, the parameter space of the ELDOR experiments has been explored here to systematically vary the bandwidth of saturation. This is already at the cutting edge of modern DNP-EPR instrumentation. Electron spin polarization was probed (via solid-echo detection) at three different probe frequencies, marked by vertical lines in Figure 2A. To generate an ELDOR profile, the pump frequency is varied across the whole EPR spectrum, while the probe frequency is fixed. The pulse lengths and amplitudes are kept constant. The polarization at the probe frequency is normalized to its Boltzmann polarization (measured by setting the pump frequency far outside of the electron resonance frequency). The ELDOR profile is then the integrated echo intensity (representing electron spin polarization) plotted versus the pump frequency. The recorded ELDOR profiles (Figure 2B) show two distinct features: saturation (shadowed with light red) where the measured echo intensity is below the Boltzmann polarization (normalized to 1) and hyperpolarization (shadowed with light blue) where the measured echo intensity representing the electron spin polarization is greater than the Boltzmann polarization (intensity > 1). The saturation of electron spins, as measured by the depth of the dip in polarization below Boltzmann (intensity <1), is at a maximum when the pump frequency matches the probe frequency. Indeed, the experimental ELDOR profiles show signatures of saturation as reflected in the ELDOR intensity dipping below 1 when the pump is closer to the probe frequency as shown in Figure 2B. However, the most striking feature observed in the experimental ELDOR profiles is the increase in echo intensity above 1 at pump frequencies well outside the pump bandwidth, which is the manifestation of hyperpolarization of electrons. The *inverse* ELDOR experiment, in which the pump pulse is fixed in frequency and the probe pulse is scanned over the entire EPR spectrum, would be more desirable for understanding the hyperpolarization effect or even DNP.^24^ However, implementing this in practice is challenging due to standing wave interference that occurs when altering the probe frequency across the entire EPR line width. Therefore, we varied pump pulse frequencies in our experiment. Later in this text, we will delve into the origin of hyperpolarization, examining whether it arises from the same group of probed spins or if it involves an augmented number of spins that contribute to the signal through a multifrequency excitation effect.

The width of saturation and the extent and exact frequency envelope of hyperpolarization vary between different probe frequencies, which is a consequence of the intrinsic heterogeneity in coupling of the spin system, leading to a distribution of spin parameters between nonexchanging spin populations. The here observed ELDOR saturation profiles are broadened much beyond the bandwidth of the μw pump pulse used (1 MHz) due to electron spectral diffusion (eSD) and cross-talk. The extent of hyperpolarization observed above Boltzmann depends on the probe frequency. When the probe frequency is set to 193.67 GHz, maximum hyperpolarization was observed for the pump frequency with an offset of ±30 MHz from the probe frequency. Negligible hyperpolarization is observed at a probe frequency of 193.69 GHz, suggesting that the saturated electron spin population is more readily exchanging with the rest of the spin ensemble. Indeed, at this probe frequency, we observe maximum eSD as reflected in a broader saturation width in the ELDOR profile, consistent with the rapid eSD obscuring selective saturation of the heterogeneous electron spin populations needed for electron spin hyperpolarization. In fact, at a trityl concentration exceeding 60 mM, the ELDOR profile is generally much more broadened, and much reduced hyperpolarization effects are observed compared to trityl samples at 35 mM concentrations (see Supporting Information Figure 1). We can infer from these observations that *selective EPR saturation* is required to produce the hyperpolarization feature.

Why is selective saturation required for hyperpolarization? What is the physical origin of hyperpolarization in a coupled spin system? Answering these questions will be an important thrust in order to optimally utilize electron spin clusters for DNP-enhanced NMR, to potentially harness the electron spin hyperpolarization for DNP, and also for understanding the spin physics of excitation and read-out of quantum information embedded in strongly coupled electron spin systems.

The hyperpolarization effect is a clear indication of the influence of strong e–e coupling since an echo intensity above 1, i.e., above the normalized Boltzmann value, is impossible to achieve for an isolated or even weakly coupled electron spin system. If so, what is the minimum system on which we can test the requirements to produce features of electron hyperpolarization? We need to incorporate the effects of higher order terms in the density matrix. The simplest system we can start with is a dipolar coupled system of two spins, where the two electron spins carry different resonance frequencies, i.e., form an AX-like system. A frequency difference of 30 MHz between pump and probe giving rise to hyperpolarization and a small through-space exchange and dipolar coupling expected between trityl radicals justifies this approximation. To obtain enhanced electron spin polarization at a given combination of pump and probe frequencies, somehow polarization has to be transferred from one electron spin population to another. Our hypothesis is that such polarization transfer requires a Zeeman order of two coupled spins forming an AX system, so that Zeeman order can be converted to longitudinal dipolar order by selective saturation of one of the A (or X) spin doublet manifolds.

We now numerically simulate a minimal two-spin AX system (using MatLab) to test our hypothesis, and in particular to understand the effect of μw manipulations. Such a system is also known to generate longitudinal dipolar order between electron and nuclear spin in the case of the Davies ENDOR experiment.^33^ The spin dynamics of this system can be modeled by considering the Zeeman interaction of the two spins and the secular coupling between them:
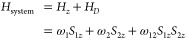
1

We ignore the effect of nonsecular e–e coupling interactions and the effect of hyperfine couplings for simplicity. The two coupled electron spins (S_1_ and S_2_) can have distinctly different Zeeman energies owing to g-anisotropy, hence constituting an AX spin system, where ω_1_ – ω_2_ > ω_12_. The Hamiltonian of a two-spin 1/2 system will have four energy eigenvalues, leading to two single quantum (SQ) transitions of each spin. The secular coupling lifts the degeneracy, so that each spin will exhibit a doublet transition, as illustrated in Figure 3A,B. Selective transitions of one of the doublet transitions are coherently induced by μw irradiation. In general, the characteristics of the observed signal depend on the quantum state of the system represented by the density matrix. The initial density matrix defined with respect to the Hamiltonian in eq 1 is given as
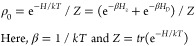
2Expanding the first few terms of the exponential, we get
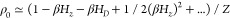
The constant term is just an identity and does not evolve under the Hamiltonian and therefore can be ignored in calculations. The β*H*_*z*_ term contains single spin order terms, *S*_1*z*_ and *S*_2*z*_, that are the Zeeman order terms. The polarization of the Zeeman order term is proportional to the factor tanh(βℏω_*i*_/2) for spin *i*, which varies between 0 (high temperature or negligible field) and 1 (low-temperature and high field). The last two terms in the density operator contain the product of *S*_1*z*_ and *S*_2*z*_ operators. These are two-spin order terms. A subtle distinction between these two terms is that the β*H*_*D*_ term is weighted by the coupling coefficient ω_12_ but (β*H*_*z*_)^2^ is simply a statistical quadratic correlation that does not have any weighting by the coupling strengths. The matrix representation of both terms is identical, and therefore they will be consistently referred to as the longitudinal dipolar order in this text, in accordance with the EPR literature.^33^ The polarization of longitudinal dipolar order is a lot smaller than that of single-spin order under high field and high temperature conditions. This is because *H*_*z*_ ≫ *H*_*D*_ by at least three orders of magnitude, and (β*H*_*z*_)^2^ is the square of the intensity of the single-spin order and therefore negligible at high temperature (i.e., if tanh(βℏω_*i*_/2) ≪ 1). Due to this reason, when simulating NMR or EPR experiments, typically only the Zeeman order term is taken into account in the initial density matrix. Substantial longitudinal dipolar order can be achieved by cooling the sample to extremely low temperatures such as liquid helium temperature for electrons and milli-Kelvin temperature for nuclei. Alternatively, it can also be achieved through hyperpolarization techniques using selective irradiation.^34^ Under our experimental condition of ∼15 K, the electron Zeeman order polarization is below 0.1, and that of longitudinal dipolar order is on the order of 0.01. Therefore, we can ignore the contribution from longitudinal dipolar order in the initial density matrix ρ_0_. However, under the effect of selective, coherent or incoherent, μw irradiation of an AX spin system, the longitudinal dipolar order and the Zeeman order become interconvertible, with the extent of conversion depending on the strength of the e–e coupling and μw irradiation parameters.^33,35^ Hence, the effect of longitudinal dipolar order cannot be ignored under ELDOR and DNP conditions (relying on monochromatic CW μw irradiation) if e–e coupling is strong. This is at the crux of the observed electron spin hyperpolarization effect observed in the ELDOR profiles. To illustrate this, we present simulations that illustrate the generation of longitudinal dipolar order under frequency-selective μw irradiation.

**Figure 3:**
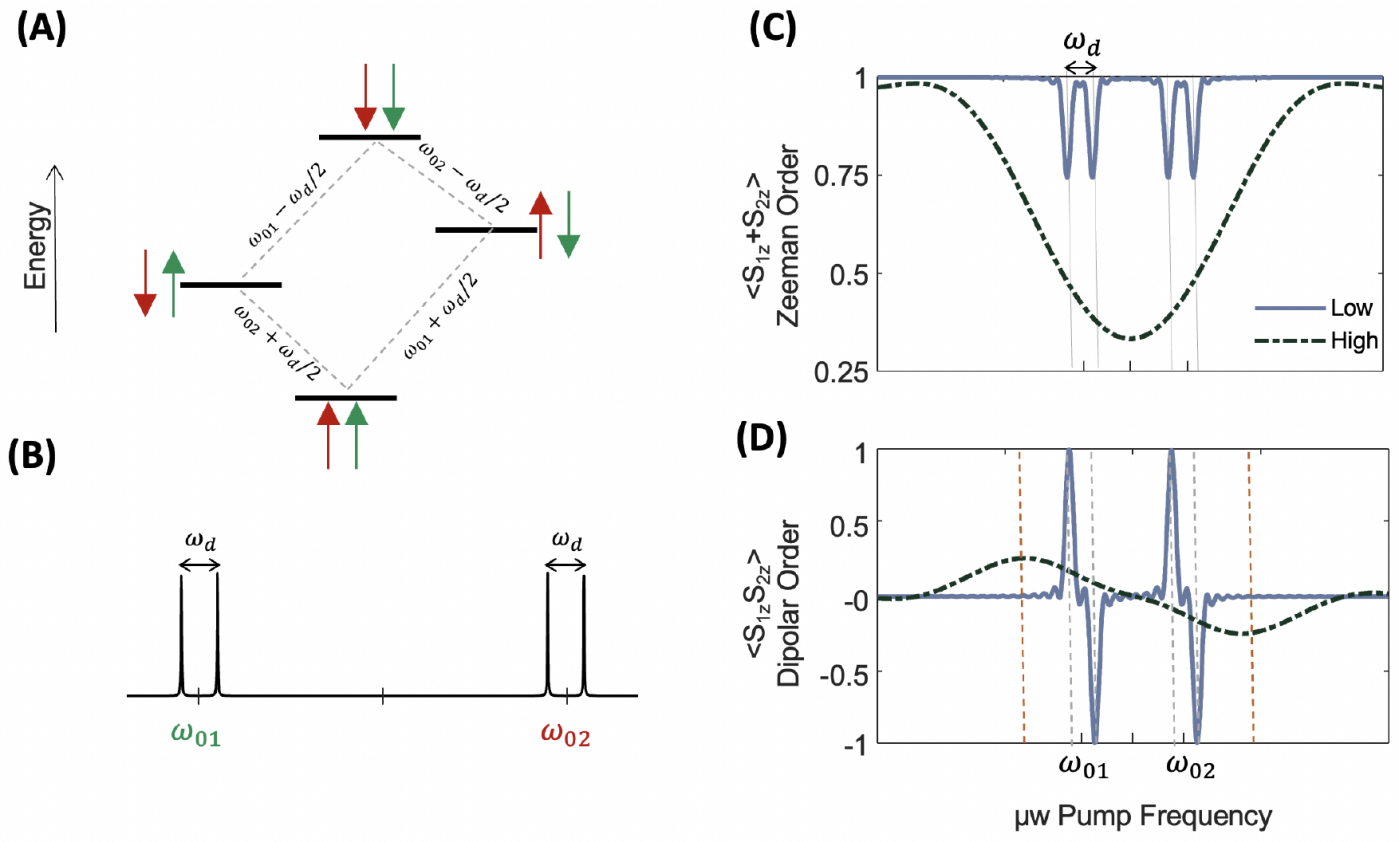
(A) Energy levels for two coupled spin 1/2 electrons. (B) Simulated single-quantum transition spectrum for the defined spin-system. (C) Simulated Zeeman-order polarization profile as a function of pump frequency swept across the spectrum for low and high powers of irradiation. (D) Simulated dipolar-order polarization profile as a function of pump frequency swept across the spectrum for low and high powers of irradiation.

Figure 3B shows that the spectrum originating from SQ transitions consists of two doublets. Each doublet shows a splitting equal to the coupling strength between the spins. ELDOR experiments are mimicked in Figure 3C and D, neglecting the effect of relaxation and electron spectral diffusion. The pump frequency is stepped through the entire EPR spectrum, and the resulting trace of *S*_1*z*_ + *S*_1*z*_ and *S*_1*z*_*S*_2*z*_ in the resultant density matrix is calculated. These traces contain the values of Zeeman and longitudinal dipolar order polarization, respectively. The normalized polarizations are plotted versus the μw pump frequency for two different μw powers. When using a low-power μw excitation, only a selective transition at the pump frequency is induced. As the pump frequency is stepped across the spectrum and matches one of the four resonance conditions in the two doublets, the saturation of the Zeeman order occurs at each of these resonances. The effect of saturation, as reflected in the reduction of *S*_1*z*_ + *S*_1*z*_ trace at the four resonances, is shown with solid lines in Figure 3C. Importantly, this process is accompanied by the creation of longitudinal dipolar order, selectively at each of these four resonances, as can be seen when detecting the *S*_1*z*_*S*_1*z*_ operator, shown with solid lines in Figure 3D. A crucial point to note is that saturation of each transition in the doublet (Figure 3C) leads to an opposite phase (sign) of dipolar order (Figure 3D). Therefore, simultaneous saturation of both the transitions of a doublet will kill the net longitudinal dipolar order. This is reflected in the simulated polarization profiles using high-power μw pump pulses. A broad-band saturation using high μw power attenuates the net longitudinal dipolar order and simply causes a broad saturation of the Zeeman order, which is an expected phenomenon. High-power irradiation maximizes the saturation of the Zeeman order and attenuates the emergence of the longitudinal order. However, even when using high-power irradiation, an unequal saturation within the dipolar manifold is possible when there is an offset in the pump frequency relative to the probe frequency. Here, the offset has to be large relative to the bandwidth of saturation of the pump pulse. This is elucidated in the longitudinal dipolar order generated upon off-resonant irradiation on either side of the multiplet (with opposite sign amplitude), as seen in Figure 3D (black dashed lines), with significant dipolar order seen at a certain offset frequency (indicated in red dashed vertical lines). These simulations show that Zeeman order polarization can be converted into longitudinal dipolar order polarization by using selective or off-resonance saturation of the coupled spins. A gradient of longitudinal dipolar order can be generated across the EPR spectrum.

Alternatively, varying the length of the saturation pulse can also control the extent of saturation via electron spectral diffusion (eSD), a process that is always operative in a coupled spin system. Hence, the efficiency of transfer to dipolar order and the optimum pump frequency where the transfer is maximized depends on the pump pulse length. This has been experimentally explored, and the resulting ELDOR profiles are shown in Figure 4A. All pulse parameters are held constant, and only the pump pulse duration is varied. For a very short pump pulse (100–250 μs), the extent of dipolar order hyperpolarization generated is far more than the saturation or reduction in Zeeman order, and hence predominantly hyperpolarization is observed (most intensity is greater than the baseline). As the pump pulse length increases (reaching ms time scale), the extent of hyperpolarization by dipolar order decreases (intensity above baseline), while the effect of saturation in Zeeman order increases gradually (intensity below baseline). Although longer pulses have a narrower bandwidth, the saturation becomes increasingly nonselective due to significant eSD effects. This is contrary to high-power pulsed EPR experiments where a longer pulse leads to narrower, more selective excitation in a pure AX spin system (lacking eSD). Moreover, the frequency offset at which maximum dipolar order is achieved increases with the length of the pump pulse. This was discussed earlier with simulated data shown in Figure 3D for two different powers, further corroborating the experimental observation (Figure 4A) and validating the simple theoretical model. In summary, nonselective irradiation attenuates the magnitude of the dipolar order polarization via self-cancellation effects.

**Figure 4:**
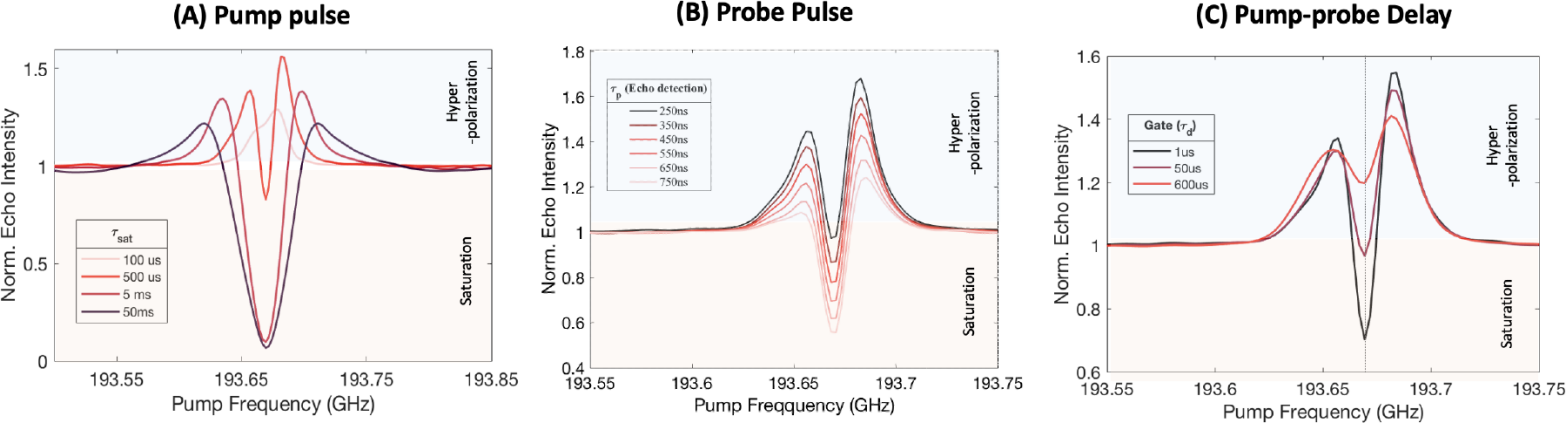
(A) Experimental ELDOR polarization profile for four different lengths of pump pulses keeping all other parameters same as in Figure 2B. (B) ELDOR polarization profile for six different lengths of probe pulses keeping the pump pulse length to 1 ms. (C) ELDOR polarization profile for three different delays between the pump and probe pulses.

We have shown that a pump pulse can change the density matrix from a pure Zeeman order (β*H*_*z*_), *S*_1*z*_ + *S*_1*z*_, to a mixture of Zeeman and dipolar order (*βH*_*D*_), *p*_1_*S*_1*z*_ + *p*_2_*S*_1*z*_ + *p*_12_*S*_1*z*_*S*_1*z*_. However, the experimental detection of the Zeeman or longitudinal dipolar order requires another μw perturbation, which converts these spin orders to observable signals or single quantum coherences. The observable signals following the perturbation have different phases for Zeeman and dipolar order. Under resonance conditions, the detectable signal of dipolar order is 90° phase shifted with respect to that of Zeeman order. Therefore, it is in principle possible to monitor the polarization of dipolar order by observing the quadrature component of the echo or FID. This principle is in fact utilized in out-of-phase ESEEM that is used to measure the coupling of electron spins in spin-correlated radical pairs in the field of solar materials to optically excited magnetic resonance.^21,36^

The flip angle of the probe pulse can also determine the relative contribution from the Zeeman versus dipolar order.^37^ The dipolar order term has faster nutation than the Zeeman order. Ideally, maximum dipolar order is obtained with a 45° probe pulse and the Zeeman order with a 90° pulse. However, this is only valid if the pulses are perfect, the μw B_1_ field is strong compared to the dipolar coupling strength, and assuming there is no inhomogeneous distribution in the B_1_ field. These requirements are not met in our experimental setup. In fact, these conditions are rarely met in high-field pulsed EPR and DNP experiments. The limitations of the low-power μw field, the large B_1_ inhomogeneity across the sample, and the presence of significant g-anisotropies in trityl radical systems make phase-sensitive detection (of in-phase and out-of-phase components) challenging. Nevertheless, we can still vary and track the relative magnitude of the Zeeman and dipolar order by simply varying the length of the probe pulse.

We test this approach by recording ELDOR profiles by varying the flip angles (durations) of the probe pulse while keeping all other parameters fixed. As can be seen in Figure 4B, the ELDOR polarization profiles clearly change with the duration of the probe pulse. For a short probe pulse, hyperpolarization (originating from the longitudinal dipolar order) dominates the ELDOR spectrum as reflected in greater intensity above the baseline, while for a long probe pulse, saturation of the Zeeman order dominates the spectrum.

The saturated Zeeman order and the longitudinal dipolar order generated with selective microwave pulses are transient in nature. Their decay back to Boltzmann equilibrium can be studied by varying the delay between the pump and probe pulses in an ELDOR experiment (pulse scheme in Figure 2B). The nonequilibrium density matrix (*p*_1_*S*_1*z*_ + *p*_2_*S*_1*z*_ + *p*_12_*S*_1*z*_*S*_1*z*_), which relaxes to the equilibrium state via various incoherent mechanisms, is not easily predicted a priori. We therefore experimentally track the recovery of the Zeeman and dipolar order signatures with time. For that, we measure three ELDOR profiles by keeping all pulse parameters fixed and only vary the delay between the pump and the probe pulse. We overlaid the three ELDOR profiles to monitor the systematic changes to their shapes (Figure 4C). To our surprise, we observe that, with an increase in the delay, the observed Zeeman order due to saturation recovers much more rapidly than the decay of the hyperpolarized signal originating from dipolar order, such that eventually only the hyperpolarization features remain dominant, even under the condition where the pump frequency is equal to the probe frequency. This suggests that the relaxation and recovery of the selectively depleted signal is faster than the decay of the hyperpolarized signal. It is possible that the hyperpolarized signal during longer delay is retrieved by conversion of dipolar order to Zeeman order under the internal Hamiltonian of the system.

Next, we investigated whether doping the trityl spin system with Gd-DOTA known to shorten *T*_1e_ relaxation can modulate the dynamics of the dipolar order. A similar ELDOR pump–probe experiment was repeated to find the condition that leads to maximum hyperpolarization as shown in Figure 5B. The echo intensity of the ELDOR experiment is plotted for 35 mM trityl doped with 2 mM GdDOTA as a function of the delay between the probe and the pump pulse after a 1 ms long τ_sat_ pulse. Such a short, low-power pump pulse can only saturate a small spin packet. The resulting saturation recovery curve again exhibited a biexponential trend, with shorter polarization recovery and decay time constants. Initially, after the pump pulse, the echo intensity dropped far below the Boltzmann intensity (indicated by the horizontal dotted lines). However, with an increasing delay between the probe and the pump pulse, the echo intensity recovered rapidly (<1 ms), possibly due to the rapid spectral diffusion between the saturated and unsaturated spins within the Zeeman order. Again, the decay rate of the hyperpolarized signal was significantly slower than the recovery rate of the depleted signal. However, the slower decay time was comparable to the *T*_1e_ relaxation time of a completely saturated EPR line of the sample (∼11 ms) as shown in the SI. We suggest that the decay of the hyperpolarized signal is marginally slower than *T*_1e_ due to mixing of Zeeman and dipolar order due to elctron spins couplings. The hyperpolarized dipolar order is transformed into Zeeman order, which decays to Boltzmann equilibrium through the *T*_1e_ relaxation mechanism. This explains why the decay rate of the hyperpolarized signal is similar to the relaxation rate of the completely saturated EPR line of the sample.

**Figure 5:**
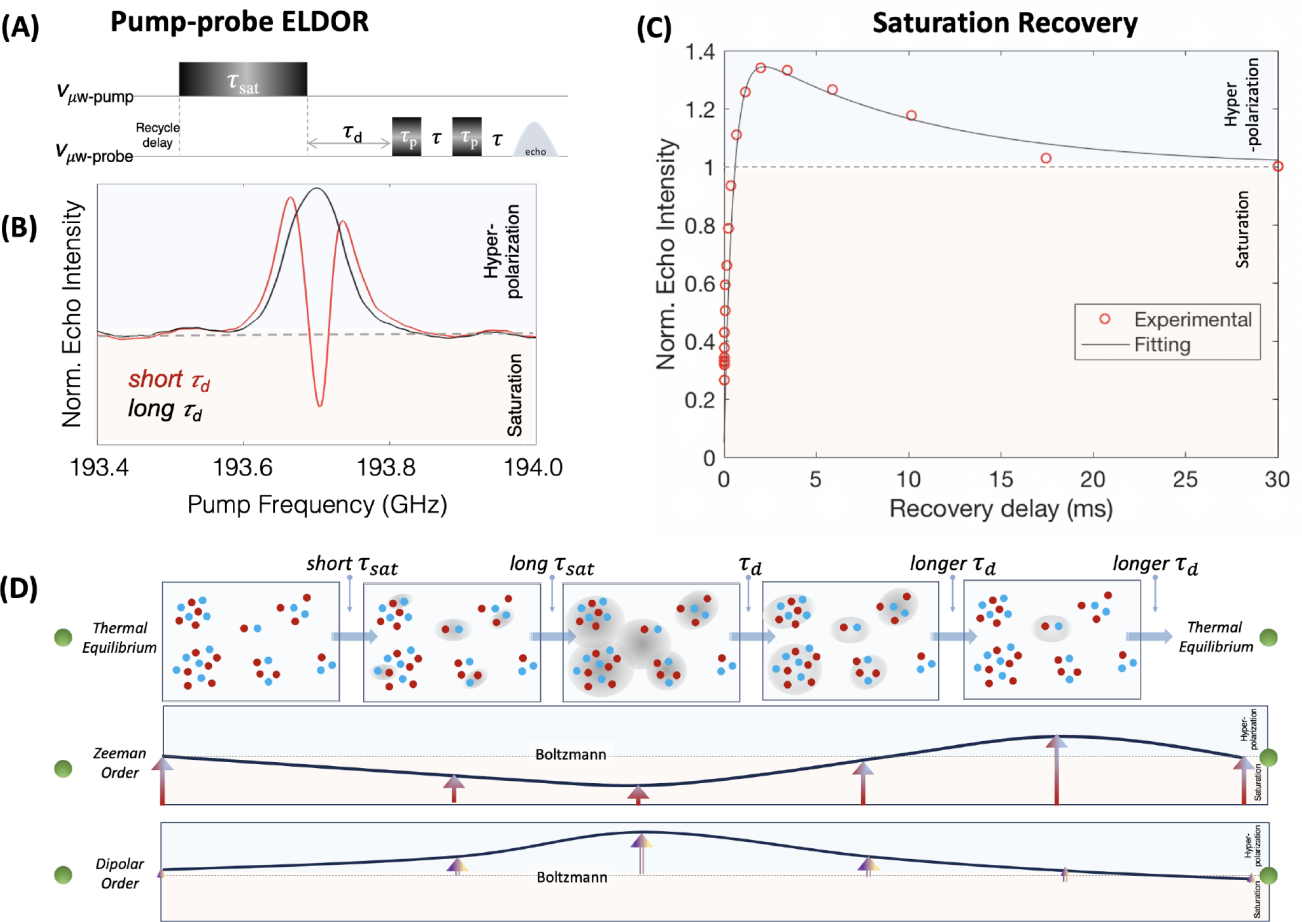
(A) ELDOR pulse schematics. (B) ELDOR profile for two different delays, τ_d_ for ∼35 mM trityl doped with 2 mM GdDOTA. τ_sat_ was set to 1 ms. (C) Magnitude of echo intensity recorded in a saturation recovery experiment for probe frequency set to 193.7 GHz. All other experimental conditions are similar to those in Figure 2. (D) Schematic illustrating the different physical processes involved during the pump–probe experiments.

It is worth making a connection to studies of nuclear spin dipolar order. The second derivative line shape of the ELDOR profile shown in Figures 2b and 4a is remeniscent of the dispersive measurements of dipolar order in nuclear spin systems.^23^ However, the just-described effect of microwave-induced hyperpolarization and the relaxation of two components with opposite sign (build-up and decay) is not common in dipolar order studies of coupled nuclear spins. The exceptions are studies and the exploitation of pure dipolar order in para-hydrogen (p-H_2_), for which the generation of hyperpolarized antiphase order by (selective) pulse excitation has been discussed. However, a detailed expose of similar effects seen in PHIP (Parahydrogen-Induced Polarization) and SLIC (Spin-Lock Induced Crossing)–SABRE (Signal Amplification by Reversible Exchange) is outside the scope of this study, especially given that pure spin order is the starting point with p-H_2_, unlike in our study where dipolar order is induced from the Zeeman order of a coupled electron spin system at thermal equilibrium. There are other significant differences between the generation of dipolar order in nuclear vs electron spin systems. In a spin system with nuclear AX configuration, ADRF-type techniques are employed to create dipolar order, as opposed to using selective irradiation. The ADRF method is generally not feasible with the pulsed EPR instrumentation currently available. Additionally, the nuclear spins are more homogeneously distributed throughout the system. In contrast, electron spin systems have significantly larger g-factor variations with orientation, known as g anisotropy, especially at high magnetic fields. These clusters are of varying sizes, while the interaction between the clusters can be weak to moderate, as illustrated in Figure 5. This results in significant heterogeneity in the microscopic makeup of the dipolar coupled spin system at hand, and a sufficiently high probability of finding AX type spin systems in clusters of spins that interact via dipolar (or exchange) interactions on the order of few to tens of megahertz^8,27,28,38^ between inequivalent spins due to their large g anisotropy. Off-resonance irradiation of the clusters during a pump pulse results in the development of (hyperpolarized) longitudinal dipolar order within each of the individual clusters and to a greater extent with longer (selective) pump pulses (illustrated in gray shades in Figure 5).^39^ It is noteworthy that Stepišnik and Slak applied Provotorov’s theory to predict the time-dependent contact between the Zeeman and the dipolar order under off-resonant irradiation of a wide NMR signal in 1973. They reported on the creation of proton dipolar order similar to the electron dipolar order in our study. However, no hyperpolarization of nuclear spins was reported or discussed in their paper.^40^

For an AX spin system, the effective spin temperatures of the different spins and their dipolar order following irradiation differ between clusters. Selective μw irradiation can drive the system out of equilibrium; spectral diffusion introduces contact between the different clustered spins (as well as more isolated spins) making the different spin temperatures more uniform. Sufficient irradiation time is needed to generate dipolar order, while longer irradiation also converts dipolar order back to Zeeman order. With a longer delay after the pump pulse that generates dipolar order, spectral diffusion continues to equilibrate the spin temperatures of the different dipolar clusters while reverting the dipolar order back to Zeeman order in a clustered spin system. Hence, the extent and timing of perturbation and observation can be controlled, which results in hyperpolarized electron spin Zeeman order (depicted with up arrows in the lower panels).

Finally, we want to draw a comparison between our report with old literature. The effects of microwave irradiation on EPR spectra have been described using thermodynamic models developed by Provotorov and Atsarkin and Rodak.^41,42^ These models employ Zeeman and non-Zeeman spin temperatures to characterize quasi-equilibrium conditions. Numerous studies have adopted this framework to demonstrate how perturbation of the Zeeman (electron or nuclear) reservoir can alter the spin temperature of the non-Zeeman reservoir. However, these studies neither address the hyperpolarization of selected electron (or nuclear) spin packets nor provide a quantum mechanical framework for detailing the microscopic, out-of-equilibrium processes during off-resonant microwave irradiation. Our study focuses on the generation of dipolar order in local spin clusters, as evidenced by frequency-selective ELDOR measurements and incorporates a purely quantum mechanical formalism to a simplified AX-type spin system, explaining the effects of selective microwave irradiation in a pump–probe scheme without the need for spin temperature formalism to describe dipolar order generation. Notably, Köckenberger and colleagues introduced the first comprehensive quantum mechanical framework for analyzing the effects of microwave irradiation in a three-electron spin system. Their research provided quantum mechanical insight into Thermal Mixing DNP, employing a minimally dipolar-coupled three-electron spin system.^29^

Next, we assessed whether the here discovered phenomenon of generating hyperpolarized dipolar order under μw irradiation is a generally applicable approach for other narrow line radicals with some intrinsic g-anisotropy. To evaluate the effectiveness of the approach developed here, we conducted tests on BDPA at a magnetic field strength of 6.9 T. The sample preparation conditions used were typical for DNP applications at high magnetic fields. We record an ELDOR profile at one given probe frequency (193.63 GHz) while varying the pump frequency, using a pump pulse duration of 250 μs and using a μw power of 350 mW (Figure 6). The experimental ELDOR profile shows a strong hyperpolarization feature in 40 mM BDPA, as observed with 35 mM trityl. Recently, there has been much interest in BDPA for its high efficiency at a high magnetic field and low μw power requirements, but the underlying mechanism is still actively debated.^7,27,43−45^ Although BDPA is expected to exhibit only SE DNP (an effect of a single electron coupling to nuclei) for ^1^H nuclei, it shows a surprisingly strong DNP enhancement when microwaves are irradiated at the center of the BDPA line, which is usually attributed to the OE effect in the literature.^43^ The OE, like the SE, is an effect that relies on a single electron coupling to nuclei, and hence does not rely on strong e–e coupling. Our ELDOR analysis of BDPA showsthe signature of strong dipolar order generated upon selective microwave irradiation, suggesting that multielectron spin effects play a crucial role in the DNP enhancement of BDPA, contrary to the commonly assumed single-electron OE effect as the main operating mechanism. Perhaps, the highly efficient DNP seen with a high concentration of trityl and BDPA type radicals is utilizing mechanisms that convert and harness transient electron spin hyperpolarization into nuclear spin hyperpolarization. However, such possibilities need to be thoroughly investigated and processes optimized that might give rise to new, effective DNP mechanisms that require only selective and lower power microwave irradiation schemes. Achieving DNP by utilizing multielectron and/or strongly coupled electron spin systems, via the triple-flip mechanism, falls under the category of TM DNP. The old literature on the spin temperature formalism of multielectron DNP is incomplete, making it insufficient for analyzing our pump–probe experiments. A microscopic understanding and quantum mechanical modeling of the TM DNP effect will advance our predictive understanding. The quantum mechanical theory of TM DNP is currently evolving, aiming to accurately predict and model how spin systems maximize DNP effects in relevant modern experimental contexts.^29,46−48^

**Figure 6:**
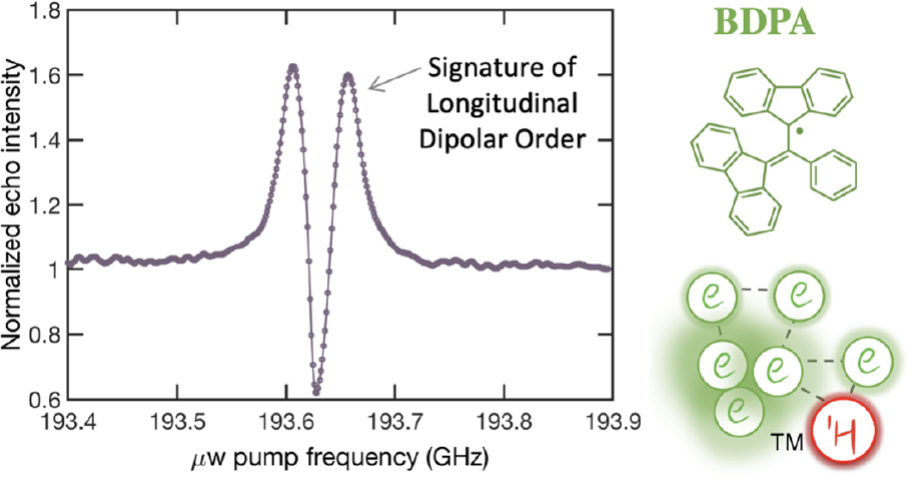
ELDOR polarization profile for 40 mM BDPA dispersed in OTP/polystyrene recorded under the same experimental conditions as in Figure 3. The hyperpolarization in the ELDOR is indicative of the clustering of BDPA molecules (right).

This study presents novel experimental signatures of coupled multielectron spin systems manifested in pump–probe ELDOR measurements that allow us to distinguish between single-electron and multi-electron effect DNP. The presence of multi-electron DNP in the trityl and BDPA systems is demonstrated through the observation of significant hyperpolarization in the ELDOR spectrum under monochromatic CW irradiation as used for DNP. The observed hyperpolarization is likely localized and transient, the effect of which can spread to the bulk through electron spin diffusion. The selective microwave (μw) saturation of an AX-spin system can first generate longitudinal dipolar order that interconverts to Zeeman order and readily gives rise to non-uniform polarization or saturation across the EPR line. To achieve non-uniform saturation with high-power or broadband μw irradiation requires a large frequency offset from the center EPR frequency. Notably, the recovery of the (selectively) saturated signal is much faster than the decay of the hyperpolarized signal. We expect there to be significant opportunities to understand and develop DNP effects arising from different spin orders. As already mentioned, PHIP is a hyperpolarization technique where the singlet order of ^1^H_2_ is transformed into nuclear Zeeman polarization through chemically induced asymmetry to give rise to enormous hyperpolarization. A similar phenomenon can be observed in the generation of both light- and/or microwave-induced dipolar order in the electron spin. This will be a subject of future investigation.

The method presented here should be applicable to a wide range of strongly coupled narrow-line paramagnetic systems with some degree of inhomogeneous broadening. This study offers a hitherto ignored potential to generate AX-like spin qubit systems for QIS and/or DNP applications by effectively controlling the polarization differential across an EPR spectrum by selective microwave irradiation of AX-like electron spin system. They include the photoactivated electron spin clusters in quantum dots or defect centers,^49^ and P1 or NV centers that have been recently shown to interact with each other via exchange coupling.^27,28^

## Supporting Information Available

EPR and ELDOR spectra of 80 mM trityl in DMSO–water, saturation recovery curve of 35 mM trityl doped with 2 mM Gd-DOTA (PDF)
