# Supplementary material for: Dipolar Order Induced Electron Spin Hyperpolarization
Source: J Phys Chem Lett. 2024 May 13;15(20):5397–406. doi: 10.1021/acs.jpclett.4c00294 (PMC11129302; doi:10.1021/acs.jpclett.4c00294)
Supplement: Supplementary file 1 — jz4c00294_si_001.pdf [file jz4c00294_si_001.pdf]

Supplementary Information Dipolar Order Induced Electron Spin Hyperpolarization

Asif Equbal ,[†,‡] Chandrasekhar Ramanathan,[¶] and Songi Han[*,§,‖]

†*Department of Chemistry, New York University Abu Dhabi, PO Box, 129188, Abu Dhabi, United Arab Emirates.*

‡*Center for Quantum and Topological Systems, New York University Abu Dhabi, PO Box, 129188, Abu Dhabi, United Arab Emirates*

¶*Department of Physics and Astronomy, Dartmouth College, Hanover, NH 03755, USA.*

§*Department of Chemistry and Biochemistry, University of California, Santa Barbara, Santa Barbara, California 93106, United States.*

‖*Department of Chemical Engineering, University of California, Santa Barbara, Santa Barbara, California 93106, United States.*

E-mail: songi.han@northwestern.edu

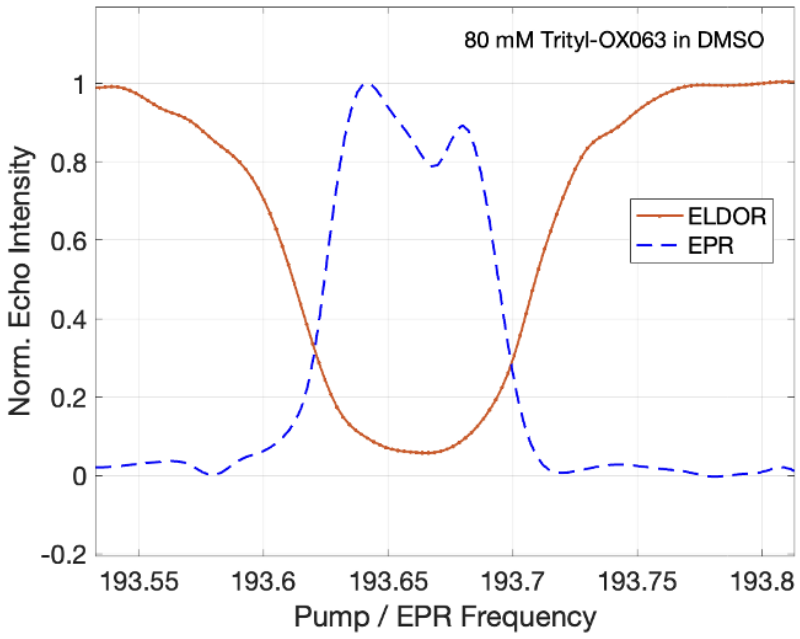

Figure 1: Experimental EPR lineshape of 80 mM Trityl in DMSO-Water solvent recorded using the shown pulse scheme at 15 K. ELDOR polarization profile for a select probe frequency. Fast electron spectral diffusion leads to a broad a saturation profile.

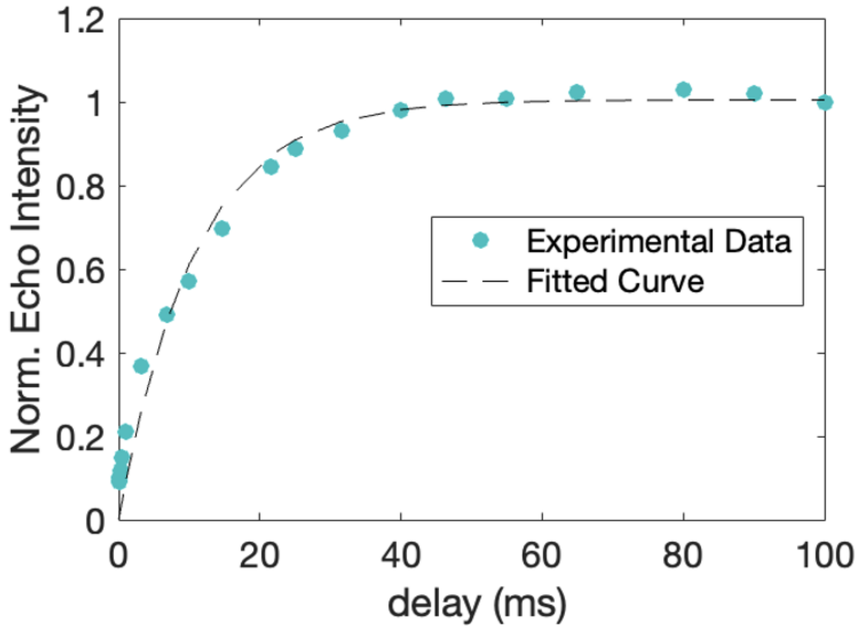

Figure 2: Saturation recovery of $\sim$ 35 mM Trityl doped with 2 mM Gd-DOTA in DMSO-Water solvent at 15 K, following a long saturation pulse at 193.63 GHz.
